# Cell Type-Specific Transcriptional Control of Gsk3β in the Developing Mammalian Neocortex

**DOI:** 10.3389/fnins.2022.811689

**Published:** 2022-03-23

**Authors:** Tadashi Nomura, Hitoshi Gotoh, Hiroshi Kiyonari, Katsuhiko Ono

**Affiliations:** ^1^Developmental Neurobiology, INAMORI Memorial Building, Kyoto Prefectural University of Medicine, Kyoto, Japan; ^2^Laboratory for Animal Resources and Genetic Engineering, RIKEN Center for Biosystems Dynamics Research, Kobe, Japan

**Keywords:** neocortex, Wnt signaling, Gsk3β, promoter, neurogenesis, evolution

## Abstract

Temporal control of neurogenesis is central for the development and evolution of species-specific brain architectures. The balance between progenitor expansion and neuronal differentiation is tightly coordinated by cell-intrinsic and cell-extrinsic cues. Wnt signaling plays pivotal roles in the proliferation and differentiation of neural progenitors in a temporal manner. However, regulatory mechanisms that adjust intracellular signaling amplitudes according to cell fate progression remain to be elucidated. Here, we report the transcriptional controls of *Gsk3*β, a critical regulator of Wnt signaling, in the developing mouse neocortex. *Gsk3*β expression was higher in ventricular neural progenitors, while it gradually declined in differentiated neurons. We identified active *cis*-regulatory module (CRM) of *Gsk3*β that responded to cell type-specific transcription factors, such as Sox2, Sox9, and Neurogenin2. Furthermore, we found extensive conservation of the CRM among mammals but not in non-mammalian amniotes. Our data suggest that a mammalian-specific CRM drives the cell type-specific activity of *Gsk3*β to fine tune Wnt signaling, which contributes to the tight control of neurogenesis during neocortical development.

## Introduction

The mammalian neocortex is a remarkable architecture in terms of its enormous tangential expansion of surface areas and a six-layered laminar organization (Nieuwenhuys, 1994). These characteristics of the neocortex are mainly accomplished by the massive production of neural progenitors during embryogenesis, which eventually give rise to a vast number of neurons and glial cells. Spatial and temporal control of neural progenitor dynamics is crucial to generate a precise number of these differentiated cells, which are prerequisites for the establishment of neocortical architectures (Lui et al., 2011; Taverna et al., 2014; Borrell, 2019).

The anatomical features of the neocortex are unique in extant mammalian species. Several comparative studies have identified neocortical homolog in non-mammalian vertebrates, despite extraordinary divergence in their size and morphology (Striedter, 2005; Nomura et al., 2014; Briscoe and Ragsdale, 2018; Cardenas and Borrell, 2020; Garcia-Moreno and Molnar, 2020). For example, the reptilian dorsal cortex (DC) is thought to be a homologous structure of the mammalian neocortex, although the DC exhibits limited tangential expansion and comprises a three-layered structure with a small number of neurons, compared to the mammalian neocortex (Ulinski, 1990; Nomura et al., 2014). Recent studies have elucidated species-specific dynamics of neural progenitors underlying morphological variations in neocortical homolog (Nomura et al., 2013; Garcia-Moreno and Molnar, 2015; Cardenas et al., 2018). However, the corresponding regulatory mechanisms that coordinate the timing of neural progenitor proliferation and differentiation are not fully understood.

Wnt signaling plays crucial roles in the regulation of neural progenitor dynamics (Clevers and Nusse, 2012). Canonical Wnt signaling is activated by the binding of secreted Wnt ligands to Frizzled receptors, which triggers the stabilization and nuclear translocation of β-catenin. In the developing mammalian neocortex, activation of Wnt signaling promotes the self-renewal of neural progenitors, which contributes to the tangential expansion of the neocortex (Chenn and Walsh, 2002). Glycogen synthase kinase 3β (Gsk3β) is a serine-threonine kinase that negatively regulates canonical Wnt signaling by facilitating the degradation of the β-catenin complex. Gsk3β is expressed in the developing neocortex in a cell type-specific manner associated with Wnt signaling inputs (Hur and Zhou, 2010). The disruption of Gsk3α and β resulted in the hyperproduction of neural progenitors and enormous expansion of the neocortex, resembling the phenotype of constitutively active β-catenin signaling (Kim et al., 2009; Ma et al., 2017). Furthermore, several studies clarified multiple roles of Gsk3β in the developing neocortex *via* the regulation of the FGF-, Notch-, and Shh-dependent signaling pathways (Jia et al., 2002; Espinosa et al., 2003; Shimizu et al., 2008; Jin et al., 2009). Spatial and temporal controls of Gsk3β activation is thus central to modulating the tempo of neurogenesis; however, regulatory mechanisms for the cell type-specific controls of Gsk3β in the developing neocortex have not been determined.

Here, we report the transcriptional controls of *Gsk3*β in the developing mouse neocortex. We identified a *cis*-regulatory module (CRM) for murine *Gsk3*β that responds to cell type-specific transcription factors (TFs), such as Sox2, Sox9 and Neurogenin2. Furthermore, we found extensive conservation of the CRM among mammals but not in non-mammalian amniotes. These data suggest that the mammalian-specific CRM drives cell the type-specific activity of Gsk3β to fine tune Wnt signaling, which contributes to the tight control of neurogenesis during neocortical development.

## Results

**FIGURE 1:**
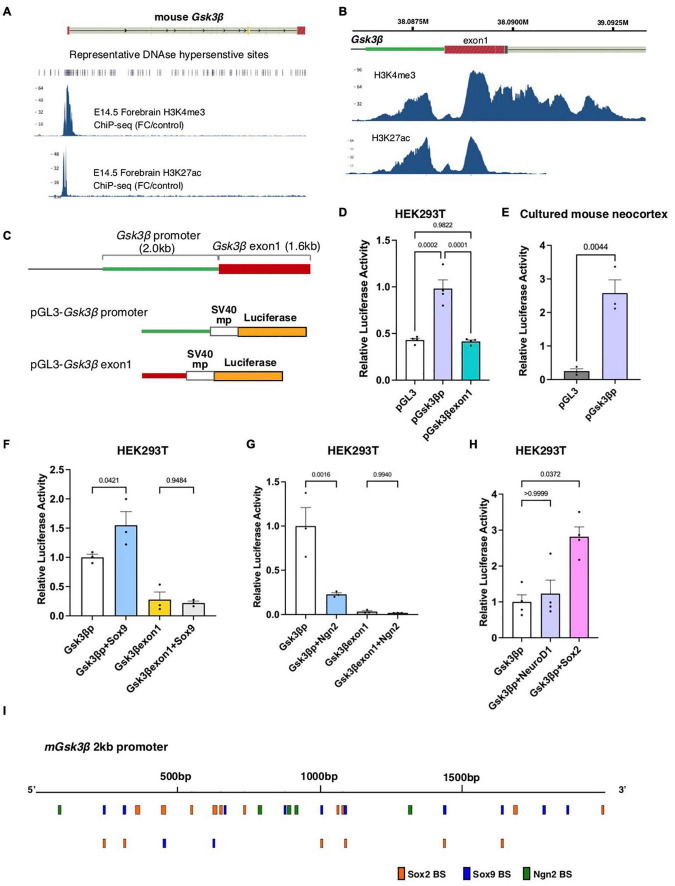
The *Gsk3*β promoter region acts as a CRM in response to cell type-specific transcription factors. **(A,B)** Histone ChIP-seq data of mouse E14.5 forebrain tissue focusing on the *Gsk3*β genomic locus (ENCODE). The peaks represent H3K4me3 and H3K27ac enrichment. **(C)** Schematic drawings of the *Gsk3*β promoter and exon 1 and reporter vectors containing these genomic fragments. **(D,E)** Luciferase activities of reporter vectors in HEK293T cells **(D)** and cultured mouse neocortex **(E)**. **(F–H)** Luciferase activities of reporter vectors containing the *Gsk3*β promoter or exon 1 in response to cell type-specific transcription factors, including Sox9 **(F)**, Neurogenin2 [Ngn2, **(G)**], NeuroD and Sox2 **(H)**. **(I)** Putative binding sites of Sox2, Sox9, and Ngn2 in mouse *Gsk3*β promoter region. All graphs represent mean + s.e.m.; *n* = 3 or 4 independent samples. Statistical analyses were performed by ordinary one-way ANOVA **(D,F,G)**, unpaired *t*-test **(E)**, and Kruskal-Wallis test with Dunn’s multiple comparison’s test **(H)**. *P*-values are represented in each graph.

Previous studies have shown that the expression of *Gsk3*β is higher in ventricular progenitors but gradually declines according to neuronal differentiation (Li et al., 2012; Ma et al., 2017). To investigate the regulatory mechanisms underlying the dynamic expression of *Gsk3*β in the developing neocortex, we searched for active *cis*-regulatory regions responsible for *Gsk3*β expression in the mouse embryonic neocortex by using the ENCODE database. Histone ChIP-seq analysis focusing on the *Gsk3*β genomic region in the E14.5 mouse forebrain indicated the prominent enrichment of H3K4me3 and H3K27ac, landmarks of open chromatin, around the putative promoter region of murine *Gsk3*β (Figure 1A). We identified two peaks of enrichment, corresponding to the 5′ UTR close to the open reading frame and the center of exon 1 (Figure 1B). Consistently, a previous study indicated that 2kb upstream region of *Gsk3*β contains C/EBP consensus sequences (Jin et al., 2020). To determine whether these genomic regions act as CRMs for *Gsk3*β expression, we isolated genomic fragments corresponding to these two peaks (designated as the *Gsk3*β promoter and exon 1) and examined transcriptional activities by luciferase assay (Figure 1C). Compared to control vector (pGL3), pGL3-*Gsk3*β promoter, but not exon1, significantly increased luciferase activity in HEK293T cells (Figure 1D). Increased luciferase activity by pGL3-*Gsk3*β promoter was also confirmed in cultured embryonic mouse brains (Figure 1E), suggesting that this genomic region acts as a CRM in the developing mouse brain. Next, we asked which TFs are responsible for the regulation of *Gsk3*β expression. We focused on several TFs that play critical roles in self-renewal or differentiation of neural progenitors and introduced expression vectors of these TFs into HEK293T cells together with reporter vectors containing *Gsk3*β promoter or exon1. We found that Sox9, a sox family protein that regulates progenitor maintenance, significantly increased reporter activity driven by the *Gsk3*β promoter, while Neurogenin2 (Ngn2), a transcription factor responsible for neuronal specification, decreased *Gsk3*β promoter-dependent transcriptional activity (Figures 1F,G). In contrast, we did not detect any responses of the reporter vector containing *Gsk3*β exon 1 in the presence of these TFs (Figures 1F,G). To further analyze the role of cell type-specific TFs in the regulation of *Gsk3*β expression, we examined the transcriptional activity of the *Gsk3*β promoter in the presence of NeuroD1 or Sox2, which play prerequisite roles in neuronal specification and progenitor maintenance, respectively. Although NeuroD1 did not change *Gsk3*β promoter-dependent transcriptional activity, Sox2 significantly increased promoter-dependent reporter activity (Figure 1H). In line with the results of reporter assay, we identified that several binding sites of Sox2, Sox9, and Ngn2 in the 2kb promoter sequences of mouse *Gsk3*β (Figure 1I). These results indicated that the genomic region corresponding to the 5′UTR of *Gsk3*β has the potential to regulate Gsk3β expression in response to cell type-specific TFs.

**FIGURE 2:**
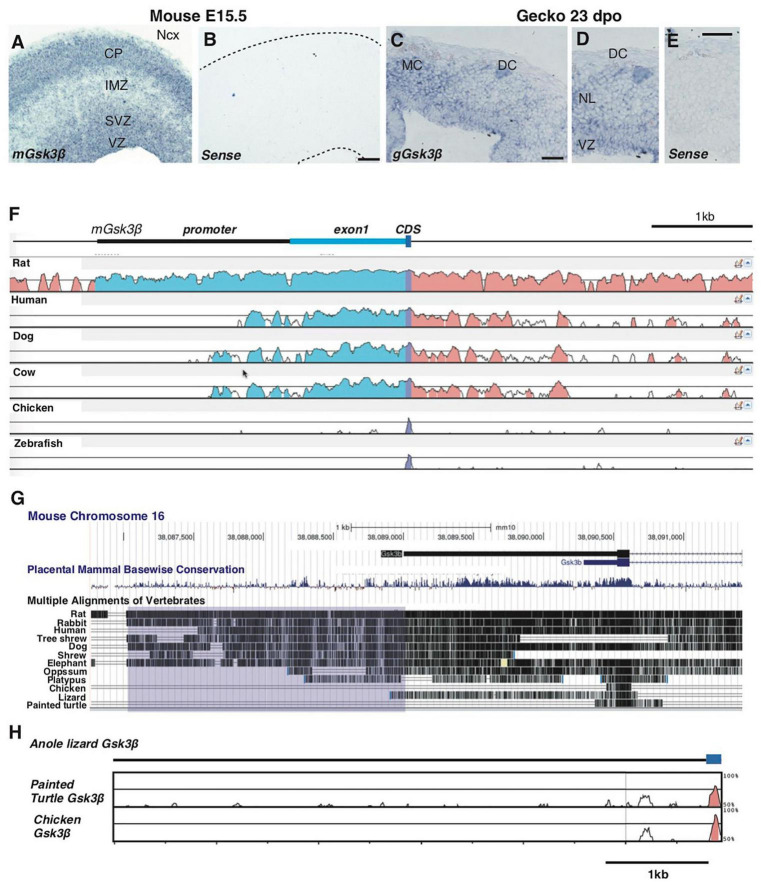
Species-specific genomic structures of *Gsk3*β in amniotes. **(A–E)** Expression patterns of *Gsk3*β in E15.5 mouse neocortex **(A,B)** and 23 dpo gecko dorsal cortex **(C–E)**. Conservation analyses of *Gsk3*β genomic loci among representative vertebrate species [**(F)**; mVISTA] and 12 amniote species [**(G)**; UCSC conservation track]. Peaks in **(F)** and vertical lines in **(G)** represent conserved genomic regions in comparison of mouse genomic sequences (mm10). The purple box in **(G)** represents the region corresponding to the 2 kb promoter of mouse *Gsk3*β. **(H)** Conservation analyses of *Gsk3*β genomic loci among non-mammalian species. Peaks represent conserved genomic regions in comparison of anole lizard genomic sequences (Ano Car2.0.v2). CP: cortical plate; IMZ: intermediate zone; SVZ: subventricular zone; VZ: ventricular zone; MC: medial cortex; DC: dorsal cortex; NL: neuronal layer. Scale bars: 100 μm **(B)**, 50 μm **(C,E)**.

We have reported that temporal changes in Wnt activity underlie the species-differences in corticogenesis between mammals and reptiles, and these changes in Wnt activity are correlated with differential *Gsk3*β expression patterns in the developing neocortex and its homologs (Nomura et al., 2020). *Gsk3*β is intensively expressed in the developing mouse neocortex, particularly in the ventricular and subventricular zones as well as the cortical plate (Figures 2A,B). We also observed the transcript of *Gsk3*β in the developing gecko dorsal cortex, with relatively higher expression in the ventricular zone compared with neuronal layer (Figures 2C–E), as previously reported (Nomura et al., 2020). To examine whether the CRM is associated with conserved expression pattern of Gsk3β between different species, we compared genomic sequences corresponding to the 5′ UTR of *Gsk3*β from various mammalian and non-mammalian species. We confirmed that the genomic sequences of the 2 kb promoter are highly conserved among mammalian species, while corresponding genomic loci were less conserved in non-mammalian vertebrates (Figures 2F,G). Notably, comparisons of genomic sequences among non-mammalian amniotes (anole lizards, painted turtles, and chicken) revealed that 5′ UTR regions of *Gsk3*β are highly divergent among these species (Figure 2H). These data suggest that ventricular-enriched expression of Gsk3β depends on the CRM that responds to cell type-specific TFs in the developing mouse neocortex, but the CRM is not associated with conserved expression patterns of *Gsk3*β in the developing brain of non-mammalian species.

## Discussion

Gsk3β plays a crucial role in the negative regulation of canonical Wnt signaling. Temporal regulation of *Gsk3*β in developing neocortical progenitors is thus critical for balancing Wnt signaling levels, which affect the shift in cell fate from self-renewal to neuronal differentiation. Here, we found that the 2 kb promoter region is responsible for controlling *Gsk3*β expression in response to cell type-specific transcription factors, such as Sox family proteins and Ngn2, suggesting that the proximal CRM contributes to the temporal controls of Gsk3β depending on cell fate progression. Gsk3β also interacts with multiple signaling pathways through the modulation of intracellular signaling components (Beurel et al., 2015). Thus, the Sox-dependent activation of Gsk3β expression is critical for blocking the overproduction of progenitors as a safeguard for self-renewal activity. A previous study indicated that Gsk3β suppresses Ngn2 activity by post-transcriptional modulation, thereby limiting the neurogenic potential of Ngn2 at early stages of cortical development (Li et al., 2012). In addition, recent studies indicated that progressive downregulation of Wnt signaling plays critical roles in successive generation of neocortical laminar specific neurons (Vitali et al., 2018; Oberst et al., 2019). These lines of evidence suggest that negative feedback between Gsk3β and Ngn2 is also important for stage-dependent tuning of Wnt signaling in the developing neocortex.

In the present study, we identified that promoter sequences of Gsk3β are highly conserved among mammals, while corresponding genomic regions are more diversified in non-mammalian species. We have previously reported that higher Wnt signaling activity in the developing reptilian DC underlies the reptile-specific patterns of neuronal migration (Nomura et al., 2020). Deletion of Gsk3α and β resulted in the failure of radial neuronal migration and abnormal neuronal morphology during mammalian neocortical development (Morgan-Smith et al., 2014). Notably, it has been reported that *Gsk3*α gene is missing in several avian species (Alon et al., 2011), suggesting that the role of *Gsk3* genes is highly divergent among amniotes, and during the evolution of mammalian lineage, Gsk3-dependent signaling became to be prerequisite for locomotive neuronal migration, a mammalian-specific migration mode in the developing neocortex. Our data also suggest that mammalian-specific CRM is not associated with cell type-specific expressions of Gsk3β in non-mammalian species, as reported by Odom et al. (2007) that conserved expression patterns of orthologous genes frequently depend on variable bindings of TFs along unaligned regions. Further investigations are required to elucidate the temporal regulation of Gsk3β expression in the progression of corticogenesis, which will provide mechanistic insights into fine tuning of Wnt-dependent neurogenesis and the evolution of mammalian-specific neurogenic patterns in the developing neocortex.

## Limitations

Although we confirmed the responsiveness of *Gsk3*β promoter in HEK293T and cultured developing mouse brains, we could not visualize activities of the promoter in neural progenitors and differentiated neurons in the neocortex. Regarding phylogenic analyses of *Gsk3*β, currently Madagascar ground gecko is only species in which the expression pattern of *Gsk3*β has been confirmed. However, genomic information of 5′ UTR of gecko *Gsk3*β has not been available, due to incomplete alignment of corresponding genomic region. To circumvent these obstacles, it is necessary to establish transgenic animals that faithfully recapitulate CRM-dependent *Gsk3*β expression, together with functional validations of *cis* and *trans* regulatory elements in other non-mammalian species, which await future studies.

## Methods

### Animals

Pregnant mice (*Mus musculus*, Slc:ICR background, 3 months) were obtained from Japan SLC, Inc., The day on which vaginal plugs were found at midday was considered embryonic day 0.5 (E0.5). All experimental procedures in this study were approved by the experimental animal committee of Kyoto Prefectural University of Medicine and Institutional Animal Care and Use Committee of RIKEN Kobe Branch and were performed in accordance with the relevant guidelines (M23-272, M23-273, and M25-102).

### Functional Genomics

ENCODE Encyclopedia version 5^1^ was utilized to evaluate histone mark enrichment at the genomic locus of mouse *Gsk3*β (mm10) based on H3K4me3 and H3K27ac ChIP-seq data of *Mus musculus* C57BL/6 E14.5 forebrain tissue (ENCSR172XOZ and ENCSR320EEW, respectively). Searching TF binding sites in *Gsk3*β promoter sequences was performed by using JASPAR CORE.^2^ Comparative analysis of *Gsk3*β genomic loci was performed using VISTA-point^3^ and the UCSC genome browser based on mouse assembly (GRCm38/mm10; chr16: 38086,769-38091,481). Multiple sequence alignment between green anole (Ano Car2.0.v2; Chr1: 423474-431390), painted turtle (Chrysemys picta bellii-3.0.3.1, Chr1: 17187654-17194971), and chicken (GRCg6a; Chr1: 80745006-80751245) was performed by using mVISTA.^4^ Five primes UTR sequences of mouse *Gsk3*β and predicted TF binding sites are deposited to Mendelay Data (doi: 10.17632/8d3t3frxb9.2).

### Plasmids

Expression vectors used in this study were constructed by using the In-Fusion HD cloning system (Clontech). Genomic fragments corresponding to the mouse *Gsk3*β promoter and 1st exon were chemically synthetized (BEX) and subcloned into the pGL3-promoter vector (Promega). Mouse *Sox9* (NM_011448) cDNAs were obtained by PCR amplification and subcloned into the pCAGGS vector (Niwa et al., 1991). pCAG-Sox2-IRES-Neo vector was a gift from Dr. Shinya Yamanaka [Addgene plasmid #13462, (Tokuzawa et al., 2003)], pCAG-NeuroD1iresGFP vector was a gift from Dr. Connie Cepko [Addgene plasmid #45025 (Cherry et al., 2011)]. pCAG-Ngn2 was a gift from Dr. Ohtaka-Maruyama.

### Luciferase Assay

HEK293T cells were transfected with the pGL3, pGL3-Gsk3β promoter or pGL3-Gsk3β exon 1 by using Lipofectamine 2000 (Thermo Fisher Scientific). To monitor the transfection efficiency, the pRL-SV40 vector expressing Renilla luciferase was transfected under all experimental conditions. To examine the response of *Gsk3*β promoter and exon1 to TFs, we introduced luciferase reporter vectors together with pCAG-Sox9, pCAG-Sox2-IRES-Neo, pCAG-Ngn2, or pCAG-NeuroD1iresGFP. As a control condition, pCAGGS empty vector was co-transfected with reporter vectors. Twenty-four hours after transfection, luciferase activity was examined with a single-tube luminometer (GL-220, MICROTEC). Firefly and Renilla luciferase activities were measured with a Dual-Luciferase Reporter Assay System (Promega). To examine transcriptional activities of reporter vectors in the developing mouse brain, we isolated E13.5 mouse forebrains and injected DNA solution containing luciferase reporter vectors into the lateral ventricle. Square pulses (28V, 50ms on-time, 4 times) were applied to brains by using an electrode (CUY650P3, BEX) and a pulse generator (CUY EDIT II, BEX). After electroporation, the brains were transferred to glass bottles filled with Dulbecco Modified Eagle Medium (DMEM) containing 10% fetal bovine serum and cultured in the rotating culture system (IKEMOTO RIKA) at 37 C° for 24 h supplied with 40% oxygen.

#### *In situ* Hybridization

*In situ* hybridization was performed according to previous studies (Ding et al., 2005). cDNAs for making RNA probes of mouse *Gsk3*β (NM_019827) were obtained from Origene and subcloned into pBluescriptSK vector. cDNA corresponding to Gecko *Gsk3*β (comp702104_c0_seq1, Reptiliomix) was chemically synthesized. The *in situ* hybridization data were obtained from samples examined in our previous study (Nomura et al., 2020), but new representative images were captured from different sections that have not been published previously.

### Statistical Analysis

For statistical analysis of the luciferase assay, three or four independent biological samples from each experimental group were compared. Normality of each data set was confirmed by Shapiro-Wilk test, and statistical significance was examined by unpaired *t*-test or ordinary one-way ANOVA with Šidák’s multiple comparisons test. For data sets that include non-gaussian distribution, Kruskal-Wallis test with Dunn’s multiple comparisons test were performed. All statistical analyses were performed by using Microsoft Excel (ver 16.54, Microsoft) and Prism9 (ver 9.3.1., GraphPad). Raw data obtained in this study is available as a Souse Data File at Mendelay Data [NOMURA, TADASHI (2022), Cell type-specific transcriptional control of Gsk3β in the developing mammalian neocortex. doi: 10.17632/8d3t3frxb9.2].

## Data Availability Statement

The datasets presented in this study can be found in online repositories. The names of the repository/repositories and accession number(s) can be found in the article/supplementary material.

## Ethics Statement

The animal study was reviewed and approved by the Satoshi Ohtshuka (Kyoto Pref Univ Med), Yasuhide Furuta (RIKEN).

## Author Contributions

TN contributed to the experimental design, data collection, interpretation, and manuscript preparation. HK contributed to the establishment and maintenance of the Madagascar ground gecko colony. HG and KO contributed to the acquisition of the reagents and interpretation. All authors discussed the data and interpretation and confirmed the final version of the manuscript.

## Conflict of Interest

The authors declare that the research was conducted in the absence of any commercial or financial relationships that could be construed as a potential conflict of interest.

## Publisher’s Note

All claims expressed in this article are solely those of the authors and do not necessarily represent those of their affiliated organizations, or those of the publisher, the editors and the reviewers. Any product that may be evaluated in this article, or claim that may be made by its manufacturer, is not guaranteed or endorsed by the publisher.

## Footnotes

1
https://www.encodeproject.org

2
https://jaspar.genereg.net

3
https://pipeline.lbl.gov/cgi-bin/gateway2?selector=vistapoint

4
https://genome.lbl.gov/vista/mvista/submit.shtml
